# Influence of Glutinous Rice Raw Material Characteristics on the Aroma Profile of Rice Wine

**DOI:** 10.3390/molecules30163315

**Published:** 2025-08-08

**Authors:** Yue Wang, Kangjie Yu, Xiongjun Xiao, Jianxia Tan, Rui Liao, Cong Li, Siyu Li, Nian Liu, Yi Ma

**Affiliations:** 1 College of Bioengineering, Sichuan University of Science and Engineering, Yibin 644000, China; 2 Sichuan Engineering Technology Research Center for Liquor-Making Grains, Yibin 644000, China; 3 State Key Laboratory for Crop Stress Resistance and High-Efficiency Production, College of Agronomy, Northwest A&F University, Yangling 712100, China; 4 Department of Sichuan Food Fermentation Industry Research and Design Institute Co., Ltd., Chengdu 611130, China

**Keywords:** rice wine, glutinous rice, key aroma compounds, sensory, gas chromatography–mass spectrometry

## Abstract

Rice wine is a beverage rich in flavor, but the flavor difference caused by rice raw materials has received little attention. To determine the key aroma compounds in rice wine, four types of samples were analyzed by gas chromatography–mass spectrometry (GC–MS), gas chromatography–olfactometry (GC-O), and sensory evaluation. Thirty-eight aroma compounds were detected in the experiment, thirteen of which were identified and quantified using the internal standard method. Additionally, multivariate statistical analyses such as partial least squares discriminant analysis (PLS-DA) effectively revealed three major differential aroma components in rice wine (VIP value ≥ 1). Furthermore, by correlation analysis, it was found that starch and fat in the raw material properties of glutinous rice were significantly and positively correlated with the main differential volatile aroma components in rice wine (*p* < 0.05). Combined with principal component analysis (PCA), the selection of glutinous rice varieties associated with starch and lipid characteristics during the rice wine brewing process is conducive to improving the overall quality of rice wine.

## 1. Introduction

Rice wine, a traditional Chinese fermented beverage, is esteemed for its delicate and harmonious flavor profile. Flavor is a crucial aspect of rice wine, influencing consumer acceptance and quality assessment [1]. The brewing climate, technology, and raw materials are key sources of rice wine flavor. Understanding their impact on aroma formation is essential for producing high-quality rice wine [2,3].

In recent years, increasing attention has been paid to the influence of raw materials on rice wine. Glutinous rice is often used as a high-quality raw material for fermentation due to its strong water absorption, high amylopectin content, and easy pasteurization and enzymatic degradation after steaming [4,5]. Under the action of microorganisms (e.g., molds and yeasts) during fermentation, macromolecules such as starch, fat, and protein in glutinous rice are broken down into small-molecule aroma precursors, including monosaccharides, fatty acids, and amino acids [6]. Consequently, varietal differences inevitably affect rice wine flavor formation. Research had proven that in experiments with rice wine made from different raw materials, those produced from ingredients with higher fat content had exhibited a higher total ester content [7]. The microorganisms had utilized the proteins in glutinous rice to produce metabolites, which had enhanced the flavor of the rice wine. Using gas chromatography–olfactometry–mass spectrometry (GC-O-MS) combined with sensory analysis, the researchers had demonstrated that the nitrogen compounds derived from protein degradation had determined the quality of the rice wine [8]. Additionally, inherent aromatic substances in glutinous rice contribute unique flavor characteristics, which are further transformed during fermentation to form complex aroma profiles [2,9]. Although existing studies have investigated the influence of glutinous rice characteristics on rice wine flavor, they have not comprehensively elucidated how key components (e.g., starch, protein, and lipids) quantitatively affect flavor compound formation. Furthermore, the integration of physicochemical parameters with sensory evaluation remains limited, making it difficult to validate the actual contribution of raw material composition to flavor development.

To clarify the influence of glutinous rice properties on the sensory flavor profile of rice wine, this study employed four distinct glutinous rice varieties for fermentation. Advanced analytical techniques, including gas chromatography–mass spectrometry (GC–MS), gas chromatography–olfactometry (GC-O), partial least squares discriminant analysis (PLS-DA), and quantitative descriptive analysis (QDA) conducted by a trained sensory panel, were used to characterize the flavor compounds in the resulting rice wines. The objective is to systematically investigate the relationship between the physicochemical properties of glutinous rice and the sensory attributes of rice wine, thereby providing a scientific foundation for targeted flavor modulation and quality enhancement.

## 2. Results and Discussion

### 2.1. Glutinous Rice Parameter Analysis

**Figure 1:**
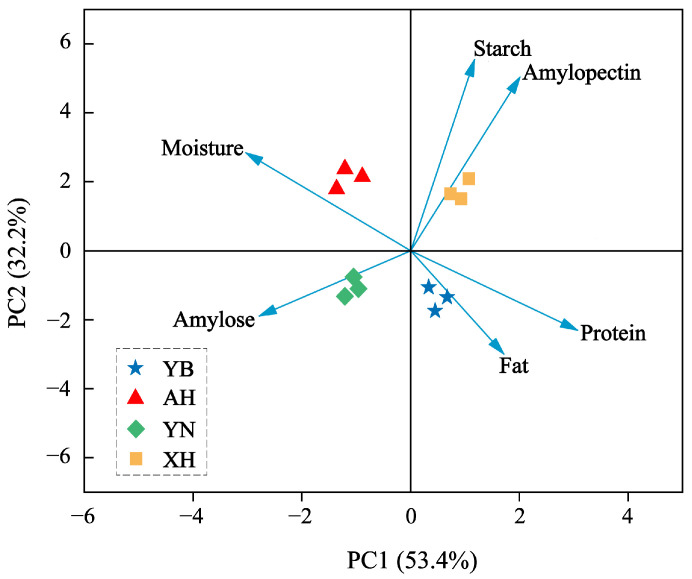
Principal component analysis (PCA) map of nutrient components of glutinous rice. XH: Guizhou Xianghe glutinous rice; AH: Anhui glutinous rice; YN: Jiangsu Yangnong glutinous rice; YB: Yibin glutinous rice.

An excessively high moisture content, exceeding 15.5%, in glutinous rice significantly elevates the risk of mold contamination and substantially compromises the quality of rice wine [10]. Table 1 showed that the moisture content of the four glutinous rice varieties ranged from 10.51% to 15.02%, which met the storage requirements for glutinous rice. During production, microbial degradation of fats produced unsaturated fatty acids, aldehydes, and ketones, enhancing flavor. However, excessive fat led to rancidity [11]. Among the four glutinous rice varieties, XH had the highest fat content at 0.92%. Starch was crucial for alcohol fermentation and sensory characteristics and was divided into amylose and amylopectin. Amylose had limited water absorption, making it less favorable for production, while amylopectin better promoted microbial growth and the formation of rice wine flavor [12,13]. XH had the highest amylopectin content and the lowest amylose content. The proteins in the raw materials contribute to the formation of rice wine flavor during fermentation through microbial metabolism. Since YB glutinous rice has the highest protein content, it may produce a more diverse range of flavor compounds [14]. To better visualize the differences in raw material characteristics among the glutinous rice varieties, principal component analysis (PCA) was applied to analyze their physicochemical properties. Figure 1 showed that the four glutinous rice varieties were distributed across four quadrants, indicating differences in their composition. YB was positioned closer to fat and protein, suggesting a correlation with these components. XH was located near starch, indicating its association with starch content.

### 2.2. Rice Wine Parameter Analysis

The basic physicochemical properties of the rice wines are presented in Table 2. The sweet fermentation starter used in the experiment contained *Rhizopus oryzae* as the predominant strain, supplemented with a small amount of yeast. Although *Rhizopus oryzae* can produce a limited amount of alcohol-producing enzymes, its ethanol-producing capacity is restricted, resulting in a thin-bodied wine. Co-fermentation with yeast can increase alcohol yield and produce a more mellow flavor. Additionally, differences in amylose and amylopectin content in glutinous rice raw materials lead to variations in starch conversion rates during enzymatic hydrolysis by microbial strains, consequently affecting alcohol production [15,16]. The alcohol content of the four rice wine groups ranged from 12.13% to 13.21%, complying with the rice wine alcohol content standard specified in NY/T 1885–2017 [17]. The soluble solids content serves as one of the critical quality indicators for rice wine, significantly influencing its flavor profile [18]. Among the four experimental groups, the soluble solids content ranged from 4.57 to 5.05 g/100 g, with AH rice wine exhibiting the lowest value. Rice wine quality is substantially affected by raw material composition. The tendency of amylose to undergo retrogradation results in reduced soluble extract content [19]. Sugar content serves as another crucial parameter for evaluating rice wine quality, mainly metabolized into ethanol and other two-carbon compounds through the acetoin pathway [20].

The reducing sugar content of the four rice wine groups ranged from 21.32 to 31.43 mg/mL. During fermentation, yeasts utilized fermentable sugars for propagation and growth, resulting in a progressive decrease in total sugar content [21]. Moderate acidity plays a crucial buffering and harmonizing role in rice wine, while also facilitating the gradual formation of ester compounds. No statistically significant differences in acidity levels were observed among the four experimental rice wine groups.

### 2.3. Results of GC–MS and GC-O

Volatile flavor compounds are the main source of rice wine aroma and one of the main factors affecting the quality of rice wine products. In this study, the volatile compounds of rice wine were analyzed using GC–MS, and a total of 38 volatile compounds were detected. On this basis, a tasting panel consisting of four judges with extensive tasting experience and expertise in GC-O analysis performed olfactory assessment of the rice wine samples, described the volatile aroma compounds present, and recorded the corresponding FD values by the aroma extract dilution analysis method (Table 3). From the table, it was observed that there were 13 aroma components with FD values ≥ 32, which were identified as important aroma substances in rice wine. Among them, substances such as phenethyl alcohol and 2,3-butanediol had FD values ≥ 256.

Esters and alcohols contribute to the floral and fruity aroma characteristics of rice wine such as apple, rose, and banana [22]. In addition, the harmony of acidity and aroma is a major feature of rice wine. Volatile organic acids are known to contribute to distinctive flavors in rice wine, with aromas described as acidic, or similar to cheese [23]. Research has demonstrated that although acetic acid and octanoic acid have relatively high thresholds, their influence on the aroma substances of rice wine is related to their concentrations [24]. Appropriate concentrations of acetic acid can enhance the harmony between flavor and aroma in rice wine, whereas excessive amounts may generate off-flavors such as fatty and rancid notes, consequently compromising the quality of the rice wine [25,26]. Ketone compounds themselves do not belong to flavor substances, but small amounts of short-chain carbonyl compounds can present sweet and fruity aromas [27]. Aldehydes were mainly derived from the oxidation and degradation of lipids. They exhibited fruity and nutty aromas, along with low odor thresholds, and contributed a spicy sensation to the wine body. A moderate concentration of aldehydes enhanced the overall balance and structural framework of the wine, playing a notable role in shaping its aromatic and flavor characteristics [27,28]. The volatile compounds in the four groups of rice wine were predominantly composed of esters and alcohols, which was a shared characteristic among them. These results corroborate the findings of Su J.J in their study on brown rice wine [29]. This commonality partially accounted for the similarities observed in certain aroma attributes across the four types of rice wine. However, since aroma compounds have different aroma intensities, exploring the distribution of substance species alone is not sufficient to explain the reasons for the differences in aroma attributes among the four rice wines.

### 2.4. Quantification of Aroma Compounds

**Figure 2:**
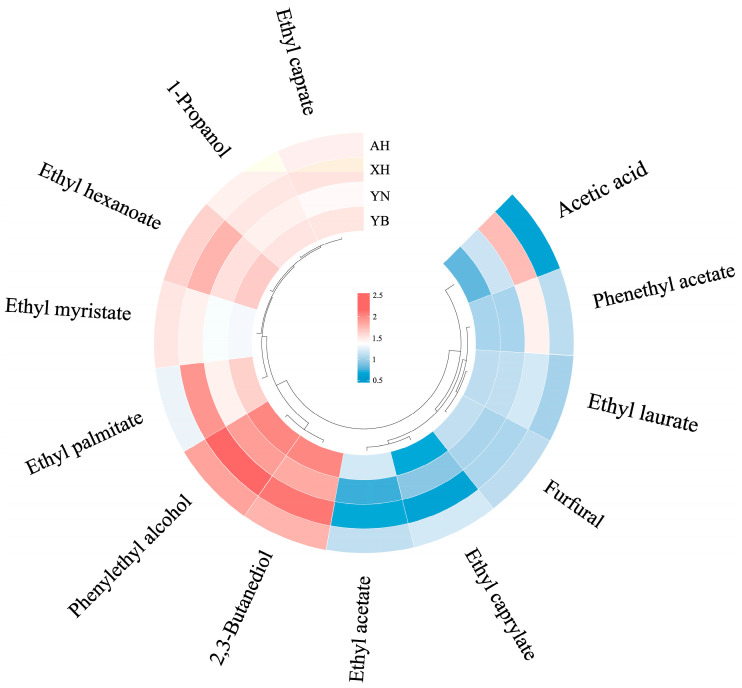
The concentration Annular heat map of 13 important aroma compounds in rice wine. Data processed by the “lg” function. XH: Guizhou Xianghe glutinous rice; AH: Anhui glutinous rice; YN: Jiangsu Yangnong glutinous rice; YB: Yibin glutinous rice.

Absolute quantification of the 13 volatile aroma substances of rice wine FD value ≥ 32 was carried out using the internal standard curve method, and the standard curves and R^2^ of the aroma substances are shown in Table 4. The contents of volatile aroma compounds are shown in Figure 2. The results demonstrate that phenethyl alcohol generally exhibited the highest concentration, followed by 2,3-butanediol. Phenylethyl alcohol increases the harmony and fullness of the wine, with an intense rose and creamy aroma [30]. Its production primarily stems from the phenylalanine metabolism pathway involving close collaboration between *Candida* and *Monascus* species [31,32]. Also, some amino acids are precursors of higher alcohols. When amino acids constitute the main nitrogen source, non-saccharomyces cerevisiae yeasts metabolize tryptophan and L-phenylalanine to tyrosol and phenylethyl alcohol via the Ehrlich pathway. These alcohols not only affect the flavor of rice wine but are also involved in the regulation of yeast growth [21]. Figure 2 shows that XH glutinous rice wine has the highest content of phenylethyl alcohol and 2,3-butanediol. A higher content of alcohols indicates their crucial role in flavor formation in rice wine. However, they generally exhibit lower odor activity values [33]. Esters are the key flavor components in rice wine, primarily formed through either the esterification of higher alcohols and fermentation-derived ethanol with organic acids, or via enzymatic synthesis mediated by *yeast* intracellular esterases [15,34]. Figure 2 demonstrates that XH glutinous rice wine has the highest content of the ethyl palmitate and the lowest content of ethyl caprylate. The esterase activity of the ethyl octanoate-producing strain declines during mid-fermentation, and its concentration progressively decreases with extended storage time [15,31]. AH glutinous rice has the highest content of ethyl caprylate, but the lowest content of acetic acid. Studies have shown that *Candida* and *Rhizopus* are positively correlated with ester content, and changes in ester content in rice wine may be related to these dominant genera [35]. Neither YN glutinous rice nor YB glutinous rice wine has lower contents of key aromatic substances. However, YB glutinous rice wine generally has higher contents of aromatic compounds than YN glutinous rice wine. This suggests that YB glutinous rice wine exhibits superior sensory intensity compared to YN glutinous rice wine [36].

### 2.5. Partial Least Squares Discriminant Analysis (PLS-DA)

**Figure 3:**
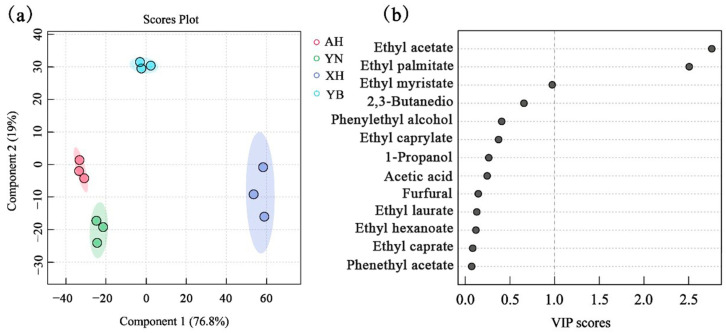
Partial least squares discriminant analysis (PLS-DA) analysis of key aroma substances (**a**) and The variable weight importance factor (VIP) of analysis of key aroma substances (**b**).

PLS-DA has strong discriminant power by projecting predictor and observed variables into a new space to build regression models. This experiment aims to further understand the flavor differences among the four samples by analyzing the quantitative data of key aroma compounds using the PLS-DA model. As shown by the results in Figure 3, the sum of the explanatory rates of the two principal components of the PLS-DA model is 95.8%, and the samples can be clearly differentiated, which indicates that the model has a good separation effect on the rice wine samples. XH glutinous rice wine and YB glutinous rice wine are clearly distinguished from each other in the model due to differences in the content of key aroma substances. The two groups of AH glutinous rice wine and YN glutinous rice wine are close to each other and show some similarity.

The variable weight importance factor (VIP) indicates the contribution of the variable to the subgroups and was used to screen for key differential compounds between the different subgroups. As shown in Figure 3, the key aroma substances with VIP values ≥ 1 are the main discriminators between sample groups and have a crucial role in distinguishing between different samples. The graph shows that ethyl acetate is the main key influencing factor, followed by ethyl palmitate. Ethyl myristate is close to 1 and may also be a key influencing factor.

### 2.6. Sensory Quantitative Descriptive Analysis

**Figure 4:**
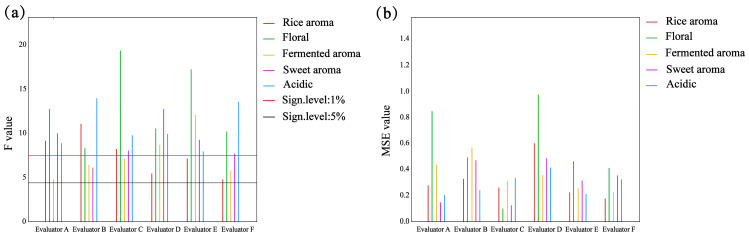
(**a**) The distinguishing ability of the evaluator. (**b**) The repeatability of the evaluator.

Based on preliminary discussions and multiple rounds of screening by the sensory panel, five sensory descriptors were ultimately selected to characterize the aromatic profiles of rice wine; namely, rice aroma, floral aroma, fermentation aroma, sweet aroma, and sour aroma. These terminologies were employed to delineate the aromatic distinctions among samples and facilitate visualization analysis. To ensure the reliability of evaluation results, Panel Check software was utilized to assess the performance of six panelists across two key dimensions: discriminative ability and repeatability. As illustrated in Figure 4a, the F-values of all six panelists for each descriptor exceeded the 5% significance level, indicating discriminative capability. Figure 4b reveals that Panelist D exhibited relatively lower repeatability in evaluating rice aroma and floral aroma, while Panelist B demonstrated weaker repeatability in assessing fermentation aroma.

**Figure 5:**
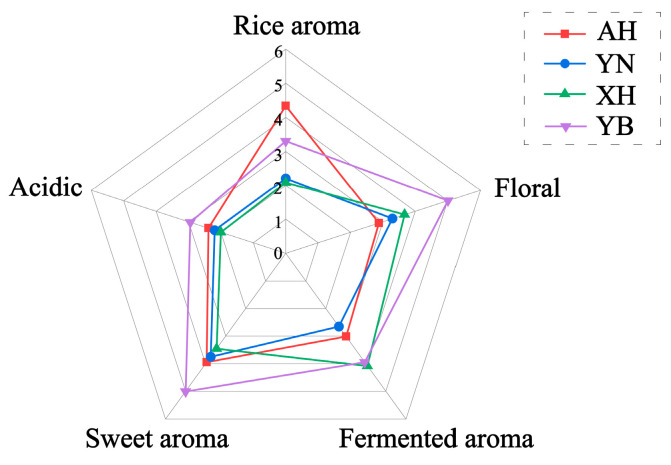
Aroma profile of rice wine. XH: Guizhou Xianghe glutinous rice; AH: Anhui glutinous rice; YN: Jiangsu Yangnong glutinous rice; YB: Yibin glutinous rice.

The characteristic aroma profiles of rice wine samples are presented in Figure 5, demonstrating significant aromatic variations among samples produced from different raw materials. During fermentation, ester compounds played a crucial role in flavor formation, particularly ethyl esters associated with subtly sweet flavor notes [1,37]. Furthermore, partial amino acids were metabolized by yeast into higher alcohols, while the remainder contributed to flavor complexity by imparting sourness and other taste characteristics [38]. Experimental results revealed that AH rice wine exhibited a distinctive rice aroma profile, primarily attributable to its elevated concentrations of esters and aldehydes and ketone compounds [39]. In contrast, YN rice wine showed relatively lower overall aroma intensity but displayed more pronounced sweet aromatic notes. The floral notes and sweetness were mainly influenced by alcohols, and YB rice wine contained relatively high levels of phenylethyl alcohol, 2,3-butanediol, and 1-propanol, which made the floral notes and sweetness of YB rice wine stand out in the aroma intensity. Notably, XH rice wine demonstrated strong fermentation-related aromas, which could be principally ascribed to the high amylopectin content in XH glutinous rice. This particular starch structure facilitated easier degradation during brewing, subsequently generating increased volatile higher alcohols (including phenylethyl alcohol and 2,3-butanediol) through side reactions—compounds known to contribute significantly to fermentation aroma characteristics [27,39].

### 2.7. Relativity Analysis

**Figure 6:**
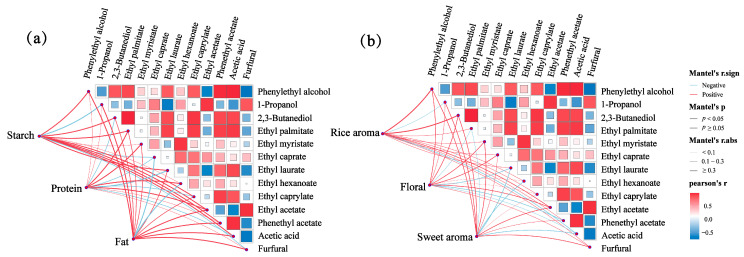
(**a**) Correlation between physicochemical and key aroma substances of glutinous rice raw materials. (**b**) Correlation between aroma profile descriptors and key aroma substances. XH: Guizhou Xianghe glutinous rice; AH: Anhui glutinous rice; YN: Jiangsu Yangnong glutinous rice; YB: Yibin glutinous rice.

The main reasons for differences in the aroma properties of rice wine are related to the nature of the raw materials and the type and content of volatile compounds produced [39]. As the nutrient contents of different glutinous rice raw materials are not balanced, the contents of the resulting aroma substances will also be different, and thus rice wine will present different sensory characteristics. The correlations between the physicochemical and key aroma substances of the glutinous rice raw materials and between the aroma characteristics of rice wine and the key aroma substances were further analyzed, as shown in Figure 6. The results show a significant positive correlation between starch content and phenethyl acetate, ethyl acetate, and ethyl palmitate, and a negative correlation with 1-propanol and furfural. Fat content is significantly positively correlated with phenethyl acetate, 1-propanol, ethyl acetate, phenethyl acetate, acetic acid, and furfural, and is negatively correlated with ethyl caprate and ethyl hexanoate. Protein content is significantly and positively correlated with ethyl acetate, ethyl palmitate, ethyl myristate, and ethyl hexanoate, and negatively correlated with 2,3-butanediol, ethyl caprate and ethyl laurate, ethyl caprylate, and acetic acid. Therefore, there is a significant positive correlation between starch and fat content and most of the key aroma substances in rice wine, and they may be important sources of aroma substances in rice wine. Rice aroma is positively correlated with phenylethyl alcohol, 2,3-butanediol, ethyl acetate, ethyl palmitate, ethyl caprate, and furfural, and negatively correlated with 1-propanol and acetic acid. There is a positive correlation between floral aroma and phenylethyl alcohol, 2,3-butanediol, ethyl myristate, ethyl caprate, and ethyl acetate, and a negative correlation with ethyl palmitate, ethyl laurate, and phenethyl acetate. Sweet aroma is positively correlated with phenylethyl alcohol, 1-propanol, 2,3-butanediol, ethyl myristate, ethyl acetate, and furfural, and negatively correlated with ethyl palmitate, ethyl laurate, ethyl hexanoate, and acetic acid.

In addition, correlations between key aroma substances can be seen in the matrix portion of the figure. There is a significant positive correlation between phenylethyl alcohol and substances such as ethyl palmitate, phenethyl acetate, and acetic acid, and a significant negative correlation with ethyl acetate and furfural. Significant positive correlation is observed between 2,3-butanediol and ethyl palmitate, ethyl laurate, ethyl caprylate, and phenethyl acetate, which may be due to their common precursor substances. Therefore, by regulating the content of various key aroma substances, the sensory modulation of rice wine can be realized.

## 3. Materials and Methods

### 3.1. Materials and Reagents

Materials: Four japonica glutinous rice varieties were used—Guizhou Xianghe glutinous rice (XH), Anhui glutinous rice (AH), Jiangsu Yangnong glutinous rice (YN), and Yibin glutinous rice (YB), supplied by Sichuan Engineering Technology Research Center for Liquor-Making Grains (Yibin, China)—and rice leaven was supplied by Angel Yeast Co., Ltd. (Yichang, China).

Solvents and reagents: petroleum ether (60~90 °C bp), ethanol (≥99.7%), anhydrous sodium sulfate (≥99.0%), hydrochloric acid (36.0~38%), sulfuric acid (95~98%), boric acid (≥99.0%), methanol (≥99.5%), iodine (≥99.8%), potassium sulfate (≥99.0%), sodium hydroxide (≥96.0%), copper sulfate (≥99.0%), lead acetate (≥99.0%), potassium sodium tartrate (≥99.0%), potassium hydrogen phthalate (≥99.0%), glucose (≥99.0%), and sodium chloride (≥99.5%) were all purchased from Shanghai Aladdin Biochemical Technology Co., Ltd. (Shanghai, China). Sec-caprylic alcohol (≥99.5%), phenylethyl alcohol (≥99.0%), 1-propanol (≥99.8%), 2,3-butanediol (≥97.0%), ethyl palmitate (≥99.0%), ethyl myristate (≥99.0%), ethyl caprate (≥99.0%), ethyl laurate (≥98.5%), ethyl hexanoate (≥99.0%), ethyl caprylate (≥99.5%), ethyl acetate (≥99.0%), phenethyl acetate (≥99.5%), acetic acid (≥99.8%), and furfural (≥99.5%) were all purchased from Shanghai Yuanye Biotechnology Co., Ltd. (Shanghai, China). C6-C26 n-alkane were purchased from Tanmo Quality Inspection TMstandard (Changzhou, China).

### 3.2. Determination of Chemical Properties of Glutinous Rice and Rice Wine

The hulled glutinous rice was crushed using a pulverizer, passed through a 40-mesh sieve, and then placed into a bag.

The moisture content was determined according to GB 5009.3-2016 (National Food Safety Standard—Determination of Moisture in Foods), using the direct drying method [40]. Approximately 2 g of the test sample was weighed into a pre-dried and constant-weight crucible, then dried for 1 h. After drying, the crucible was cooled in a desiccator for 0.5 h before weighing. This process was repeated until the difference between two consecutive weighings was ≤2 mg, indicating that a constant weight had been achieved. The final moisture content was calculated based on the mass difference before and after drying.

The fat content was determined according to GB 5009.6-2016 (National Food Safety Standard—Determination of Fat in Foods), using the Soxhlet extraction method [41]. A 2 g test sample was weighed and placed in a filter paper. After adding petroleum ether, tightly packed and sealed for reflux extraction over 8 h. Following extraction, the sample was dried, cooled to room temperature, and weighed. This procedure was repeated until constant weight was achieved.

The starch content was determined according to GB 5009.9-2016 (National Food Safety Standard—Determination of Starch in Foods), using the acid hydrolysis method [42]. A 2 g test sample was weighed and defatted by washing with petroleum ether and ethanol to remove lipids and soluble sugars. The sample was then transferred to a conical flask, mixed with 30 mL of 50% hydrochloric acid, and subjected to boiling water reflux condensation for 2 h. After cooling, 2 drops of methyl red indicator solution were added, and the solution was adjusted to neutrality. Subsequently, 20 mL of 200 g/L lead acetate solution and 100 g/L sodium sulfate solution were added and mixed thoroughly. The mixture was transferred to a 500 mL volumetric flask, diluted to volume, and filtered. The filtrate was titrated until the blue color just disappeared, marking the titration endpoint. The final starch content was calculated using formula.

The protein content was determined in accordance with GB 5009.5-2016 (National Food Safety Standard—Determination of Protein in Foods) using the Kjeldahl method [43]. A 2 g test sample was subjected to alkaline distillation to liberate ammonia, which was then absorbed in a boric acid solution (20 g/L, *w*/*v*). The absorbed ammonia was titrated with a standard 0.05 mol/L sulfuric acid solution, and the nitrogen content was calculated based on the acid consumption. The protein content was subsequently derived by multiplying the nitrogen content by the conversion factor.

The amylose content was determined in accordance with GB/T 15683-2008 (Rice—Determination of Amylose Content) [44]. The method is based on the iodine-binding property of starch, where amylose forms a characteristic blue complex with iodine. Sample pretreatment involved removing interfering components (primarily amylopectin) using methanol solution. The purified sample was completely gelatinized in a boiling water bath, then reacted with iodine reagent to develop the blue amylose–iodine complex. The absorbance of the complex was measured at 720 nm using a spectrophotometer.

The determination of sugar content is performed as follows [45]. Transfer 10 mL of the sample to a 500 mL volumetric flask, add 50 mL of water and 5 mL of 6 mol/L hydrochloric acid. Heat the mixture in a 65 °C water bath for 15 min, then cool it. Subsequently, add 2 drops of 1 g/L methyl red indicator solution and neutralize with 200 g/L sodium hydroxide solution until the red color disappears. Dilute to the required volume with water and filter the solution for analysis. Pipette 5 mL each of Fehling’s solution (Solution A and B) into a 250 mL conical flask, add 30 mL of water, and heat to boiling. Introduce 2 drops of methylene blue indicator, then titrate with the prepared sample solution until the endpoint is reached.

Alcohol content determination is conducted as follows [46]. Transfer 50 mL of the sample into a 500 mL distillation flask, add 100 mL of water, and distill until the distillate volume reaches 100 mL. Take 1 mL of the distillate in a test tube, add 0.5 mL of 0.0167 mol/L potassium dichromate solution and 2 mL of 98% sulfuric acid (95~98%). Heat the mixture in a boiling water bath for 10 min, then cool to room temperature. Measure the absorbance at a wavelength of 600 nm.

Determination of pH meter: transfer 10 mL of the well-mixed sample and measure the pH value using a calibrated pH meter.

The determination of soluble solids is conducted as follows [29]. Pipette 10 mL of the sample and dilute with an equal volume (50% *v*/*v*) of distilled water. Measure the soluble solids content of the test solution using a handheld refractometer.

### 3.3. Rice Wine Brewing

The shells of 500 g of glutinous rice grains were removed. The rice was rinsed twice with 700 mL of pure water to remove the unremoved, obviously black, and broken grains. After adding 700 mL of pure water, it was soaked at 20 °C for 12 h. After soaking the glutinous rice, the water was drained through gauze and steamed in a steamer until the glutinous rice had no white center and was not soft or mushy. The steamed glutinous rice was then poured out and spread out to cool down to 30 °C to 32 °C. The sweet wine yeast was then mixed with the glutinous rice at 0.35% of the yeast mixing amount. The glutinous rice mixed with yeast was then put into the sterilized fermentation tank, with the center hollowed to leave a hole and the lid sealed with water, and placed in a constant temperature incubator at 28 °C for fermentation for 5 days.

### 3.4. GC–MS Analysis

The method of volatile substance detection was referred to the method of Zhou with slight modification [47]. Headspace solid-phase microextraction (HS-SPME) was used to extract the aroma components. In a 15 mL headspace vial, 8 mL of the wine sample was added, followed by the precise addition of 10 μL of the internal standard sec-caprylic alcohol at 50 μg/mL, and 2 g of sodium chloride was added. After the wine sample was preheated at 45 °C for 5 min, the aged microextraction fiber was inserted into the headspace vial. Then, the fiber was extended to a position 1.5 cm above the sample liquid surface and adsorbed for 30 min. After adsorption, the fiber was retracted and quickly transferred to the GC injection port, where it was thermally desorbed at 250 °C for 3 min. The GC–MS investigation used an Agilent 8890 GC (Agilent Technologies, Palo Alto, CA, USA) with 5975C MS (Agilent Technologies, Palo Alto, CA, USA).

Chromatography was performed on a DB-WAX capillary column (30 m × 0.25 mm i.d., 0.25 μm film thickness; Agilent Technologies, Inc.). The inlet temperature was set at 250 °C, and the non-split injection mode was used. The heating program was as follows: initial temperature set at 40 °C, maintained for 5 min; 2 °C/min heating to 60 °C; 5 °C/min heating to 180 °C, maintained for 5 min; 10 °C/min heating to 230 °C, maintained for 10 min. The carrier gas was high purity He (99.99%) with a constant flow rate of 1.2 mL/min and no shunt. Electron Ionization (EI) was performed with an electron energy of 70 eV. The acquisition mode was full scan, with a scanning range of 35–500 amu and an ion source temperature of 230 °C. The analysis was conducted three times.

### 3.5. Aroma Extract Dilution Analysis (AEDA) and GC-O Analysis

The 50 mL rice wine sample was diluted to 10% vol with ultrapure water and saturated with NaCl. In a separatory funnel, the sample was extracted three times with 210 mL of redistilled dichloromethane (70 mL × 3). Each extraction was performed on a shaker at 400 rpm for 10 min. After extraction, the organic phases were combined, dried overnight with anhydrous sodium sulfate, filtered, and then concentrated using a rotary evaporator at 45 °C with a rotation speed of 70 rpm. The concentrate was stored at −20 °C, with three parallel experiments conducted for each sample.

The rice wine aroma extract was serially diluted with redistilled dichloromethane at a 1:2 ratio. Each team member performed one GC-O analysis for each dilution until no aroma could be detected. The highest dilution at which an aroma compound could be detected was recorded as its flavor dilution (FD) factor. To minimize experimental error, only compounds detected by at least two panelists in the same sample were recorded.

An Agilent 7890A GC system with sniffer port ODP 4 (GERSTEL, Shanghai, China) and DB-WAX capillary column was used for the GC-O analysis. The GC-O column oven was set up with a ramp-up program consistent with the GC–MS analysis, with the sniffer port at 230 °C. Four panelists with more than 10 years of tasting experience were trained for two weeks in a controlled test environment according to the ISO 8589 (2010) specifications, and upon completion of the training, the aroma compounds in rice wine were evaluated through olfactory analysis and characterization using the AEDA method.

### 3.6. Qualitative and Quantitative Analysis of Volatile Compounds

The volatile aroma components were characterized by mass spectrometry (MS) and retention index (RI), combined with GC-O assistance. The RI represents the relative retention time of target metabolites compared to adjacent n-alkanes. Mass spectral characterization was performed by searching and comparing with NIST/Wiley Database, and the identification results with a match of more than 80% were retained. Quantification of volatile aroma components was carried out by the internal standard curve method for absolute quantification.

### 3.7. Sensory Evaluation Panel

For this step, we referred to the method of Huang Ling [48]. Twenty-five individuals, evenly split between males and females and aged 22 to 26, were chosen from the Sichuan Brewing Special Grain Engineering Technology Research Center for olfactory experiments and quantitative descriptive analysis (QDA). These participants underwent an 8-week training program, with two sessions per week, each lasting 1.5 h. The training covered fundamental theories of sensory evaluation, aroma identification of rice wine, aroma description training, and aroma intensity ranking exercises. Following (“Sensory Analysis—General Guidance for the Selection, Training, and Management of Assessors—Part 1: Selected Assessors.”) for sensory analysis [49]. Following rigorous evaluation, 10 panelists were selected to form the tasting panel for olfactory assessment of rice wine samples. For testing, 10 mL aliquots of each wine sample were dispensed into 30 mL tasting cups, randomly coded and covered with lids. Samples were presented sequentially in randomized order, with each panelist performing triplicate evaluations. A 5–10 min interval was maintained between sample assessments to ensure olfactory sensitivity and evaluation accuracy. The panel leader organized group discussions to refine and consolidate aroma descriptors, ultimately establishing a consensus-based lexicon of quantitative aroma attributes. Reference standards corresponding to each descriptor were selected for panelist training. Intensity calibration was conducted using a 7-point scale (0 represents the weakest and 6 represents the strongest), with specific aroma reference materials detailed in Table 5. Training and qualification assessments were performed in a controlled sensory evaluation environment maintained at constant temperature, with adequate lighting and odor-free conditions. Panelists were evaluated for assessment accuracy, repeatability, and panel consistency. From the initial 10 candidates, 6 panelists (3 male, 3 female) were selected to perform QDA of the rice wines. Each selected panelist conducted triplicate evaluations during formal testing.

### 3.8. Statistical Analysis

All the above experiments were set up with three replicates. Principal component analysis was used to preliminarily distinguish the differences in physicochemical properties of glutinous rice varieties. Then, analysis of variance was used to test the significance of the differences between groups and verify the homogeneity of variance. Subsequently, Duncan’s multiple range test was used to determine the significance. The data were analyzed using SPSS 17.0 software. The accuracy and stability tests of the judges were conducted using Panel Check. The PLS-DA model was used to screen key differential compounds and was visualized using the online plotting website (https://www.metaboanalyst.ca/MetaboAnalyst/ModuleView.xhtml (accessed on 8 January 2025)). The model was evaluated through cross-validation. The correlation analysis was performed using R software, with Pearson correlation coefficients calculated. The remaining charts were created using Origin 2024 software. All statistical analyses were based on *p* < 0.05 as the significance criterion. The aroma descriptions in Table 3 were derived from FEMA (Flavor Ingredient Library (https://www.femaflavor.org/flavor-library (accessed on 10 February 2025)) and QDA. All aroma characterizations have been verified by our panel of experts.

## 4. Conclusions

In this experiment, the physicochemical indexes of four kinds of glutinous rice were determined, and the volatile compounds of rice wine were qualitatively and quantitatively analyzed by GC–MS. On this basis, the aroma profile of the rice wines were comprehensively evaluated using the sensory quantitative description method. Quantitative analysis showed that alcohols and esters were the main flavor components in rice wine, and their contents were higher than those of other flavor compounds. Phenylethyl alcohol and 2,3-butanediol were the most abundant volatile flavor compounds in the test group, with the highest content in XH glutinous rice wine; the lowest content was in acetic acid compounds in AH glutinous rice wine. Differences in aroma composition among the samples were analyzed using the PLS-DA method, which showed that ethyl acetate, ethyl palmitate, and ethyl myristate were the main differential compounds distinguishing the different rice wine samples (VIP value ≥ 1). The aroma characteristics of the rice wines were further analyzed by the quantitative sensory descriptive method. Results demonstrated that YB glutinous rice wine exhibited the most prominent floral and sweet aroma attributes, while XH glutinous rice wine showed the weakest sour aroma intensity. Combined with correlation analysis, this showed that there was a significant positive correlation between fat and starch content and most of the key aroma substances, such as phenylethyl alcohol and phenethyl acetate. Combining the results of the multidimensional analysis, YN glutinous rice wine was found to have a prominent sweet aroma. YB glutinous rice had a correlation with proteins and fats and a relatively high content of alcohols, which made the floral and sweet aroma of YB glutinous rice wine stand out. XH glutinous rice had a correlation with starch and has the highest content of phenylethyl alcohol and 2,3-butanediol, which made the fermentation aroma stand out. AH glutinous rice wine had a relatively high content of esters like ethyl myristate and a relatively low content of substances that caused off-flavors such as acetic acid, so the rice aroma is prominent.

This experiment provides important references for the quality control of rice wine products and the selection of brewing-specific grains. In the future, it can focus on combining microbiomics and targeted metabolomics technologies to study the intrinsic mechanisms and the impact of microorganisms on the formation of rice wine flavor throughout the process. In addition, a complete fermentation kinetics detection system can be established, thereby providing more scientific basis for the research on the selection of glutinous rice raw materials and the improvement of rice wine quality.

## Figures and Tables

**Table 1:** Basic physicochemical indexes of glutinous rice (%).

Sample	Moisture	Starch	Amylose	Amylopectin	Protein	Fat
YN	13.54 ± 0.02 b	70.62 ± 0.46 c	2.15 ± 0.08 a	68.33 ± 0.26 c	7.58 ± 0.09 b	0.87 ± 0.03 ab
AH	15.02 ± 0.22 a	75.83 ± 0.06 b	1.48 ± 0.07 b	74.35 ± 0.10 b	7.53 ± 0.26 b	0.64 ± 0.02 c
XH	10.77 ± 0.12 c	78.32 ± 0.04 a	1.26 ± 0.08 c	77.05 ± 0.11 a	8.36 ± 0.09 a	0.92 ± 0.05 a
YB	10.51 ± 0.08 d	69.50 ± 0.02 d	1.27 ± 0.07 c	68.23 ± 0.08 c	8.58 ± 0.04 a	0.82 ± 0.07 b

Values with different letters (a–d) in a column are significantly different using Duncan’s multiple comparison tests (*p* < 0.05). XH: Guizhou Xianghe glutinous rice; AH: Anhui glutinous rice; YN: Jiangsu Yangnong glutinous rice; YB: Yibin glutinous rice.

**Table 2:** The content of chemical component of sweet rice wine.

Sample	Alcohol Content (%)	Soluble Solids (g/100 g)	Total Sugar (mg/mL)	pH
YN	12.31 ± 0.04 c	5.02 ± 0.01 a	21.32 ± 0.47 c	3.51 ± 0.13 a
AH	13.05 ± 0.12 b	4.57 ± 0.35 b	28.04 ± 0.98 b	3.38 ± 0.03 a
XH	13.21 ± 0.08 a	5.01 ± 0.01 a	28.95 ± 0.62 b	3.54 ± 0.09 a
YB	12.13 ± 0.04 d	5.05 ± 0.03 a	31.43 ± 0.35 a	3.45 ± 0.04 a

Values with different letters (a–d) in a column are significantly different using Duncan’s multiple comparison tests (*p* < 0.05). XH: Guizhou Xianghe glutinous rice; AH: Anhui glutinous rice; YN: Jiangsu Yangnong glutinous rice; YB: Yibin glutinous rice.

**Table 3:** Identification of potentially important aroma compounds in rice wine using GC–MS and GC-O techniques.

No.	CAS	Compound ^1^	Aroma Description	RI ^2^	Identification ^3^	FD factor ^4^			
						XH	AH	YN	YB
Esters									
1	628-97-7	Ethyl palmitate	Faintly waxed	2262	MS, O, RI	>256	64	128	128
2	124-06-1	Ethyl myristate	Sour	2042	MS, O, RI	128	>64	>64	>64
3	110-38-3	Ethyl caprate	Brandy, Pear	1392	MS, O, RI,	64	64	64	64
4	106-33-2	Ethyl laurate	Floral, Fruit	1835	MS, O, RI	>32	>32	>32	>32
5	627-90-7	Ethyl undecanoate	Coconut, Fruit	1652	MS, O, RI	8	8	8	16
6	123-66-0	Ethyl hexanoate	Fruit, Brandy	1241	MS, O, RI	128	>64	>64	>64
7	106-32-1	Ethyl caprylate	Fruit, Floral, Fat	1425	MS, O, RI	32	>32	32	32
8	141-78-6	Ethyl acetate	Aromatic, Grape	906	MS, O, RI	>32	>32	>32	64
9	54515-77-4	2-Octyl acetate	Fruit, Jasmine-like	1250	MS, O, RI	8	8	8	16
10	123-92-2	Isoamyl acetate	Pear, Apple	1065	MS, O, RI	4	4	4	4
11	103-45-7	Phenethyl acetate	Honey, Rose, Floral,	1940	MS, O, RI	64	32	32	32
12	28598-81-4	Ethyl 9-bromononanoate	Fruit, Rose	1900	MS, O, RI	4	4	4	8
13	6114-18-7	Ethyl elaidate;	Floral, Fruit	2345	MS, O, RI	8	8	8	8
14	123-25-1	Diethyl succinate	Cotton, Fabric	1673	MS, O, RI	4	4	4	4
15	54546-22-4	Ethyl 9-hexadecenoate	--	2167	MS, O, RI	--	--	--	--
16	111-62-6	Ethyl oleate	Dairy	1988	MS, O, RI	--	--	2	
Alcohols									
17	60-12-8	Phenylethyl alcohol	Sweet, Rose	1936	MS, O, RI	>256	>256	>256	>256
18	98-00-0	Furfuryl alcohol	Caramel	900	MS, O, RI	4	4	4	2
19	78-83-1	Isobutanol	Apple, Bitter	1095	MS, O, RI	32	32	32	64
20	71-23-8	1-Propanol	Candy, Alcohol	1023	MS, O, RI	128	128	128	128
21	513-85-9	2,3-Butanediol	Butter, Sour	976	MS, O, RI	>256	256	256	256
22	123-51-3	Isoamylol	Burnt, Floral, Malt	1204	MS, O, RI	8	8	8	8
23	57-55-6	Propylene glycol	--	950	MS, O, RI	--	--	--	--
24	764-48-7	2-(Vinyloxy)ethanol	--	1053	MS, O, RI	--	--	--	--
25	149-32-6	Erythritol	Sweet	2328	MS, O, RI	8	8	8	16
26	111-27-3	1-Hexanol	Woody, Herbaceous, Sweet	1365	MS, O, RI	8	4	8	8
27	540-51-2	2-Bromoethanol	--	954	MS, O, RI	--	--	--	--
28	598-75-4	3-Methyl-2-Butanol	Alcohol	1156	MS, O, RI	4	4	4	4
29	15356-70-4	Menthol	Peppermint fragrance	1247	MS, O, Std	2	--	--	2
Acids									
30	64-19-7	Acetic acid	Vinegar	1455	MS, O, RI	128	64	64	64
31	124-07-2	Octanoic acid	Cheese	2132	MS, O, RI	2	2	--	2
Aldehydes									
32	124-19-6	Nonanal	Fat, Herb, Floral	1394	MS, O, RI	8	4	4	8
33	98-01-1	Furfural	Almond, Burnt sugar	1482	MS, O, RI	>32	>32	>32	>32
34	75-07-0	Acetaldehyde	Fruit	744	MS, O, RI	16	16	8	16
35	620-02-0	5-Methyl furfural	Burnt sugar	1589	MS, O, RI	2	--	2	--
Ketones									
36	116-09-6	Acetol	Herb, Malt	1281	MS, O, RI	2	4	2	--
37	111-13-7	2-Octanone	Floral, Bitter, Fruity	1295	MS, O, RI	--	2	2	--
38	5878-19-3	Methoxyacetone	--	965	MS, O, RI	--	--	--	--

^1^ Aroma compounds detected in the samples. ^2^ The retention index of volatile compounds on DB-WAX columns. ^3^ Method of identification: MS—mass spectrum comparison using NIST11.L library; O—aroma descriptor; RI: identification of aroma compounds by retention indices calculated on DB-WAX. ^4^ FD was defined as the maximum dilute degree of perception for the flavor in rice wine. XH: Guizhou Xianghe glutinous rice; AH: Anhui glutinous rice; YN: Jiangsu Yangnong glutinous rice; YB: Yibin glutinous rice.

**Table 4:** Standard curves of 13 important aroma compounds in rice wine.

No.	Compound	Slope	Intercept	R^2^
1	Ethyl palmitate	164.22	−0.1089	0.9953
2	Ethyl myristate	94.06	0.3133	0.9964
3	Ethyl caprate	85.61	0.0118	0.9947
4	Ethyl laurate	124.47	0.0718	0.9945
5	Ethyl hexanoate	115.25	−0.0064	0.9905
6	Ethyl caprylate	96.87	−0.0142	0.9940
7	Ethyl acetate	51.10	−0.0032	0.9957
8	Phenethyl acetate	35.88	0.0008	0.9976
9	Phenylethyl alcohol	51.78	0.0207	0.9944
10	1-Propanol	45.61	1.5385	0.9972
11	2,3-Butanediol	94.81	0.9001	0.9947
12	Acetic acid	75.98	−0.0041	0.9963
13	Furfural	21.96	−0.0003	0.9993

**Table 5:** References of aroma descriptors.

Sensory Descriptor	Sensory Description	Reference Standard
Rice aroma	Rice-like grain aroma	100 mL of 1% aqueous ethanol containing 5 g of rice
Floral	Aroma similar to that of roses, moonflowers	1 mg/L aqueous solution of phenethyl alcohol
Fermentation aroma	Aroma similar to fermented foods such as kimchi and soy sauce	100 mL of a 10% aqueous ethanol solution of 1 mg/L 2-butanol
Sweet	Aroma like honey, mash	100 mL of a 10% aqueous ethanol solution of 200 g/L honey
Acidic	The aroma of balsamic vinegar	100 mL of a 10% ethanol aqueous solution of 1 mg/L acetic acid

## Data Availability

All data available are presented in this manuscript.
